# Shrimp allergy: clinical characteristics and food challenge outcome in a centre in the north of Portugal

**DOI:** 10.1186/2045-7022-3-S3-P72

**Published:** 2013-07-25

**Authors:** A Coimbra, L Cruz, JF Oliveira

**Affiliations:** 1 Serviço de Imunoalergologia, Centro Hospitalar São João, Porto, Portugal

## Background

Shrimp is a common food allergen and may cause serious reactions.

### Objective

A review of the clinical files of patients who were referred due to suspected shrimp allergy.

## Methods

A retrospective analysis of the clinical charts of 30 patients who were referred to our department between January 2009 and June 2010 due to suspected shrimp allergy. The study included skin prick tests (SPT) to common inhalant allergens and shrimp; skin prick-prick testes (SPPT) with shrimp, total and specific IgE (sIgE) and open food challenge tests (OFC).

## Results

The average age was 34.9±10.3 years (24-63), 24 (80%) were female. The most common clinical symptoms were cutaneous in 57% and only 2 (7%) had a history of anaphylaxis and 1 presented unspecific symptoms. Twenty-four patients underwent SPT with shrimp and 8 (33%) of them were positive including the 2 with anaphylaxis. Twenty-three patients underwent SPPT and 6 (26%) were positive including the 2 with anaphylaxis. Only these 2 patients had both positive SPT and SPPT. Fifteeen (73%) (out of 22 patients with IgE) had elevated total IgE. Specific IgE was increased in 14 (48%) with values ranging between 0.37 and 91.7KU/L. The 6 patients with positive SPPT also had elevated sIgE. Of the 26 (87%) who performed SPT, 22 (85%) were house dust mite (HDM) positive, including the 7 (87.5%) patients with positive shrimp SPT. Nineteen patients underwent OFC: 14 (74%) were negative, 3 (16%) were positive and 2 (10%) unconclusive. The 3 patients with positive OFC had negative SPPT. The 2 patients with a history of serious anaphylaxis did not undergo OFC. The cumulative doses varied between 31.1and 128.9g. The doses of the positive OFC were between 52.0 and 64.8 g. With respect to comorbid conditions, 10 (33%) had asthma and/or rhinitis; 70% were allergic to HDM and 30% were allergic to both HDM and pollens. Two of the 3 patients with positive OFC as well as the 2 with serious anaphylaxis had a history of allergic asthma and/or rhinitis.

## Conclusion

The OFC were fundamental for the clarification of the suspected shrimp allergy; only 3 were positive. This reinforces that food challenges are necessary for a correct diagnosis. All the patients with a positive OFC had negative shrimp SPPT and only one had a positive shrimp SPT. The value of total and sIgE did not appear to have any relation to the outcome of the OFC.

## Disclosure of interest

None declared.

